# In-silico structural analysis of *Heterocephalus glaber* amyloid beta: an anti-Alzheimer's peptide

**DOI:** 10.22099/mbrc.2023.48223.1862

**Published:** 2024

**Authors:** Ali Javanmard, Maryam Azimzadeh-Irani, Ghazal Tafazzoli, Ayla Esmaeilzadeh, Mohammad Shirinpoor-Kharf, Seyyed Mohammad Hasan Haghayeghi

**Affiliations:** Faculty of Life Sciences and Biotechnology, Shahid Beheshti University, Tehran, Iran

**Keywords:** Amyloid Beta, Alzheimer's Disease, Heterocephalus glaber, AlphaFold2, Molecular Docking

## Abstract

*Heterocephalus glaber*, known as the Naked mole-rat, has an extraordinary immunity to Alzheimer's disease. The pathological hallmark of Alzheimer’s disease is cerebral accumulations of plaques, consisting of self-aggregated amyloid beta peptides. *Homo sapiens* and *H. glaber* amyloid beta peptides are different in only one amino acid. Herein, computational structural analyses were carried out to determine whether plaque development in *H. glaber* is prevented by the replacement of His13 with Arg13 in the amyloid beta peptide. AlphaFold2 was used to predict the structure of the *H. glaber* amyloid beta peptide. HADDOCK and Hex were used to self-dock the peptides and dock ions on peptides, respectively. Illustrations were made by PyMol and ChimeraX. Using VMD, we calculated the radius of gyration. The phylogenetic analysis was conducted by Mega. The results showed an accurate structure with two alpha helices separated by a short coil for *H. glaber*. Self-docking of the two amyloid beta peptides demonstrated a globular conformation in the *H. glaber* dimer, implying the unlikeliness of amyloid beta peptides’ self-aggregation to form fibrillar structures. This conformational state resulted in lower electrostatic energy compared to *H. sapiens*, contributing to *H. glaber’s* lower tendency for fibril and, ultimately, plaque formation. Phylogenetic analysis confirmed that amyloid precursor protein is highly conserved in each taxon of rodentia and primata. This study provides insight into the connection between the structure of *H. glaber* amyloid beta and its plaque formation properties, showing that the Arg13 in *H. glaber* leads to fibril instability, and might prevent senile plaque accumulation.

## INTRODUCTION

Alzheimer's disease (AD) is the most widespread irreparable neurodegenerative disease in the growing population of seniors. The clinical characteristics of AD include cognitive impairment, disturbances in normal daily activities, and changes in an individual's behavior [1]. Scientists have linked this destructive disorder to amyloid beta peptide (Aβ) accumulation, tau protein malfunction, and selective neuronal loss. Aβ has an essential role in the pathogenesis of AD as a critical component of senile plaques. Aβ, a 36 to 43 amino acid peptide, is manufactured by proteolytic processing of a type 1 transmembrane protein called amyloid precursor protein (APP) which is expressed in different tissues, especially in the synapses of neurons [2]. This glycoprotein plays an indispensable role in a variety of biological activities like signaling, transportation, and neuronal homeostasis [3].

This protein comprises a single membrane-spanning domain, a large extracellular glycosylated N-terminus, and a short cytoplasmic C-terminus. APP is cleaved by enzymes β- and γ-secretase. This membrane-bound endoprotease plays a vital role in generating Aβ. Depending on the cleavage site of APP, the final amyloid peptides vary in length [4]. The abnormal and imbalanced production of Aβ from the APP and its elimination from the brain leads to Aβ accumulation in the cerebral cortex. This phenomenon leads to neurotoxicity and generates senile plaques [1].

It is believed that the abnormal function and aggregation of a protein called tau can also be related to AD pathogenesis. Aβ is thought to have an essential impact on the tau protein to malfunction [5]. Primates are known to be suitable AD models due to their phylogenetic similarity to *H. sapiens* and longevity [6-10]. APP sequence is highly homologous amongst primates, and all non-human primates express *H. sapiens* sequence Aβ. However, with few exceptions, little to no signs of tauopathy have been reported in primates [7-11]. The presence of both Aβ plaques and tau tangles is essential for full phenotypic expression of AD [6]. 

Despite biological similarities between primates, *H. sapiens* exhibit exceptional susceptibility to AD. Although non-human mammals like monkeys, apes, and dogs develop senile plaque, no evidence shows AD-like pathology and cognitive issues. It was concluded from this data that post-translational conversions may cause the unique susceptibility of *H. sapiens* to AD in conformity of Aβ fragments [6].

In contrast to primates, rodents do not naturally exhibit senile plaques as they age, and the reason might be that most of them express an Aβ sequence that differs from primates in 3 amino acid residues [12]. These three residues are all placed in the N-terminal extracellular 28-residue segment of the Aβ peptide. Other residues are mostly hydrophobic and can be found in the transmembrane domain [13]. Rats and mice are prevalent rodent AD models. While rats are easier to handle in laboratories, and their larger brain makes them more suitable candidates for surgery due to easier access, mice are known to be technically preferable as AD models [14, 15].

*H. glaber*, also known as the Naked mole-rat (NMR), is the longest-lived rodent with high cerebral Aβ levels. However, this species has no record of senile plaque formation and age-related Aβ expression increase. The *H. glaber* Aβ shows more significant homology to the *H. sapiens* Aβ sequence compared to mice and rats. The Aβ sequence in this species differs from *H. sapiens* Aβ in just one amino acid residue (H13R). Moreover, the two histidines in *H. sapiens* Aβ fragments are suggested as metal binding sites that aid self-aggregation and this substitution in the *H. glaber* Aβ sequence may lead to a reduced tendency to self-aggregation [16, 17]. 

In this work, we investigated how a single amino acid can influence structural properties and aggregation propensity. To this end, we studied whether this specific change from histidine to arginine in the Aβ sequence might lead to great conformational and structural differences, resulting in aggregation properties. We also tested this hypothesis on another rodent with high sequence homology to the *H. glaber* Aβ sequence to check if it shows immunity to plaque formation.

## MATERIALS AND METHODS

**Protein Sequence Collection: **Knowing that some other fragments cleaved from the APP also account for plaque formation, we drew our phylogenetic tree based on this protein to find the most similar proteins to the *H. glaber* APP. For this purpose, the National Center for Biotechnology Information [18]. was utilized to obtain the *H. sapiens* APP sequence with 770 amino acids, which was then used as a query to run NCBI blastp across the taxa rodentia and primata separately. The sequences were gathered from the reference proteins database (refseq_protein). The “LOW-QUALITY” and “PREDICTED” sequences from the 100 results were all removed so the remaining assessed sequences could be used. Since the sequences were substantially conserved, very high Max and Total scores were obtained. Therefore, these values could not be used as a criterion to exclude unwanted sequences. The accession numbers of all these sequences are available in Table S1 of supplementary information. (For *H. sapiens* and *H. glaber*, all isoforms were used, whereas only isoform 1 sequences of the rest of the species were utilized). 

**Sequence Alignment and Phylogenetic Analysis: **A total of 57 sequences, 30 for rodentia and 27 for primata were aligned with Mega version 11.0.13 software using the ClustalW technique [19]. Moreover, Mega was used to carry out a phylogenetic analysis based on the previously obtained sequences, with the maximum likelihood algorithm and bootstrapping with 500 replicates. Then the tree was customized and color-coded to demonstrate primata and rodentia.

**Structure Prediction, Visualization, and Alignment: **Using AlphaFold2 Colab [20-22]. the 3D structure of the *H. glaber* Aβ was predicted (sequence obtained from a previous study [23]). The template mode was set to pdb70, and the prediction ran based on PDB structures [‘7b3j_A’, ‘6shs_G’, ‘3ifn_P’, ‘2lp1_A’, ‘2loh_B’, ‘6iyc_E’, ‘6yhx_A’, ‘4mvi_B’] as templates. The multiple sequence alignment mode was set from MMSeq2 (default) to singleseq. MMSeq2 is a method that predicts the 3D structure of a protein and takes a broader approach by considering different levels of detail in protein structure which includes capturing information about larger structural patterns and the specifics of amino acids. On the other hand, singleseq method will only use the amino acid sequence of a protein and predict its 3D structure. For proteins with no known related structures, AlphaFold2 can still use singleseq mode to predict its 3D structure. This is pretty useful for proteins that are unique or poorly characterized [20, 24]. 

AlphaFold2 offers predicted Local Distance Difference Test (pLDDT) score coloring and Predicted Aligned Error (PAE) plots. The pLDDT shows how confident AlphaFold2 is about the structures in every amino acid position. Higher scores of pLDDT, show higher confidence of structure prediction. The PAE plot shows the relative alignment confidence when aligning on any residue from the predicted structure and known structures. Scores will show distance differences in Angstroms. Lower scores in PAE plots will indicate a more accurate relative position of two amino acids in a peptide. The global superposition template modeling (TM) value is a valuable statistic to qualify or quantify the structure prediction and to compare two protein structures (the model to a target or template structure). The pairwise error prediction, which is calculated as a linear projection from the final pair representation, yields the estimate of the TM-score (pTM). Moreover, the PAE plot is calculated by AlphaFold2 using pTM scores. The higher values of the pTM score mean a higher structure prediction accuracy [25]. 

Additionally, recycle number was increased to obtain a more confident structure. UCSF ChimeraX version 1.4 [26]. was used to prepare the figures and demonstrate the predicted local distance difference test (pLDDT) coloring provided by the AlphaFold2 Colab notebook [27]. For a better understanding, PyMOL 2.5.4 was used to align the AlphaFold2 output with *H. sapiens* Aβ peptide (PDB ID: 1IYT) [28]. 

To check the accuracy and reliability of AlphaFold2 performance on the prediction of our models, we further used SWISS-MODEL to predict the 3D structure of 42 amino acid Aβ [29]. PROCHECK [30] was used to calculate a Ramachandran plot of predicted 3D structures in order to compare the two servers using different approaches in structure prediction (**Fig. S1**).

**Molecular Docking: **HADDOCK 2.4 [31, 32] was used to dock the output given by AlphaFold2 and 1IYT with themselves, respectively. Moreover, Hex 8.0.0 [33, 34]. was used to dock Cu^2+^ and Zn^2+^ ion’s Spatial Data files obtained from PubChem [35]. on both structures. Hex 8.0.0 predicted the precise location of these ions on Aβ peptides obtained from AlphaFold2 prediction and *H. sapiens* Aβ peptide (PDB ID: 1IYT). PyMOL 2.5.4 was used to visualize the results, prepare figures, and align structures. Then, VMD 1.9.4a53 (Visual Molecular Dynamics) was used to calculate the center of mass and the radius of gyration to determine whether Aβ dimers form a fibrous or globular structure. The scripts used for this calculation are provided in the supplementary information [36]. 

## RESULTS

**Figure 1:**
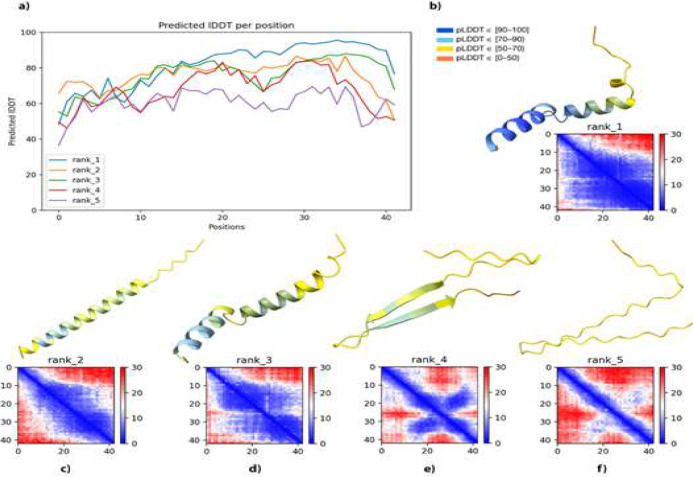
Structure prediction of the *H. glaber* Aβ protein using AlphaFold2. **a**) The pLDDT broken-line graph, shows per residue structure confidence, in this graph, all ranks are separated by colors and each line indicates one structure. The horizontal axis shows residue numbers and the vertical axis shows the pLDDT. **b-f**) Five best-predicted structures and their confidence with pLDDT colors and PAE plot. Figure legend is obtained from AlphaFold2 pLDDT coloring [32].

This study aimed to find out if there was any different and distinguishable conformational change in the 3D structures of Aβ peptides in *H. sapiens* and *H. glaber*. There was already an available structure of *H. sapiens* Aβ in PDB [PDB ID: 1iyt]. Therefore, only the structure of the *H. glaber* 42-amino acid Aβ was predicted using AlphaFold2. AlphaFold2 output provided five structures sorted from the most confident, rank 1, to the least confident, rank 5 [Fig. 1].

**Figure 2:**
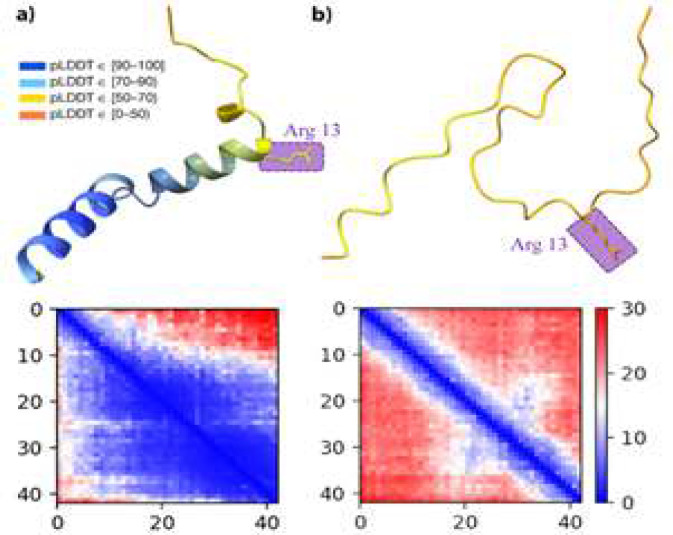
Comparison of Aβ structures and PAE plots with different MSA modes in the structure prediction process. **a) **Singleseq mode: The rank 1 structure of *H. glaber* Aβ. The Arg13 and its side chain are shown with a purple label and yellow sticks. **b) **MMseq2 mode (UniRef and Environmental): The Arg13 and its side chain are marked purple

According to pTM and mean pLDDT scores [Table S2], the rank 1 model of Aβ structure offered the overall most confident per-residue prediction [Fig. 2a]. *H. glaber* Aβ structure included two alpha helices, one more accurately predicted than the other, separated by a 3-amino-acid-long coil including Val24, Gly25, and Ser26. We want to assess the intramolecular interactions of Arg13 in Aβ and investigate the intermolecular interactions with other Aβs. Meanwhile, the PAE plot should be interpreted in a way that shows molecular prediction confidence according to this amino acid. From the PAE plot [Fig. 2a], it can be inferred that when aligning on Arg13, we can be highly confident in the range of residues Gly9 to Lys16 and almost confident on residues Phe4 to Gly9 and Leu17 to Gly37. Regarding that, we have two alpha helices (one of them located at Val12 to Asp23 and the second one at Asn27 to Ile41 of the protein sequence) and a coiled structure between these two, the relative positions of residues Arg13 and Asn27; also relative positions of residues Asp23 and Asn27 are dark blue in PAE, and this shows that the two alpha helices are placed confidently towards each other and the predicted structure can be used for molecular docking and assessment of intramolecular and intermolecular interactions [Fig. 2a]. AlphaFold2 pLDDT color scores correctly correspond to the accuracy of the predicted structure. These scores are on a scale of 0 to 100 and they show per residue confidence in the predicted structure. Hence these colors are analyzed to indicate the accuracy of AlphaFold2 prediction in our work. Rank 1 Aβ structure of *H. glaber* shows regions with relatively high to moderate pLDDT colors. From residue Asp1 to Val12, it is 70>pLDDT>50. From residue Arg13 to Lys28, the confidence is relatively high, pLDDT is between 70 and 90, and a generally good backbone prediction is observed. For residues Gly29 to Val40, pLDDT is almost above 90, and efficient for other investigations. For the two last amino acids in the structure the pLDDT decreases to the range of 70 and 90 [Fig. 1a and b].

In contrast to the rank 1 structure, others do not show a confident folding prediction [Figure 1b-f]. The pLDDT coloring method is mostly yellow and light blue, indicating 50 to 70 percent confidence. The rank 2 structure is an elongated helix in which the angle presented in rank 1 is not observable [Fig. 1c]. The rank 3 structure is quite similar to rank 1, but the angle between the two helices is displaced [Fig. 1d]. Although this structure suggests a higher length for the first helix, it depicts a less confident fold that could be due to the length suggested by the AI. Higher length would require more accuracy which is not observable. The rank 4 structure is a coiled structure showing two beta-strands forming a beta-sheet, and the area of confidence is limited to Lys17-Asp23 and Ala30-Val36 in the strands [Fig. 1e]. The rank 5 structure is a coiled structure that does not illustrate any definite conformation for the protein [Fig. 1f]. 

MMseq2 and Singleseq modes were each used separately to obtain the structures to compare their outputs and assess the performance of each in predicting the structure of the Aβ peptide [Fig. 2]. Comparing the model, we obtained from Singleseq with MMseq2, it is evident that amino acids of the Aβ sequence from MMseq2 [Fig. 2b] show low confidence (pLDDT <50) in their relative positions, and AlphaFold2 cannot determine whether this structure is helical or it forms strands. 

**Figure 3:**
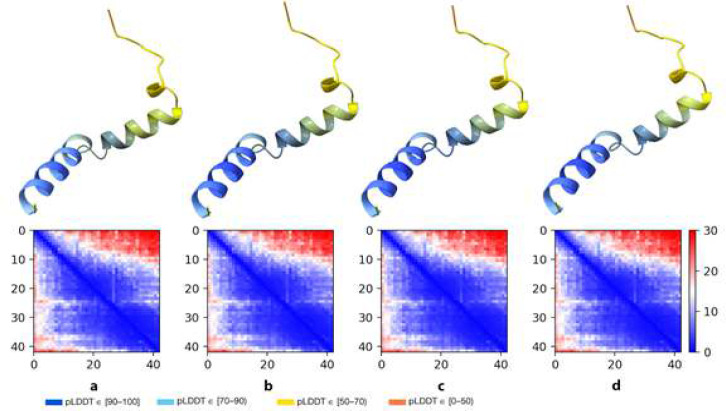
Aβ structures and their PAE plots. Obtained from AlphaFold with various recycling numbers 3, 6, 12, and 24 (**a-d**). In these structures, when increasing the recycle number, the pTM score of structures increases significantly from a to b and is the same for b, c, and d. Then the pLDDT score increases from a to c, indicating that the AlphaFold method can enhance the quality of outputs with higher recycling numbers [pTM and mean pLDDT scores are available in Table S.3 of supplementary information].

**Figure 4:**
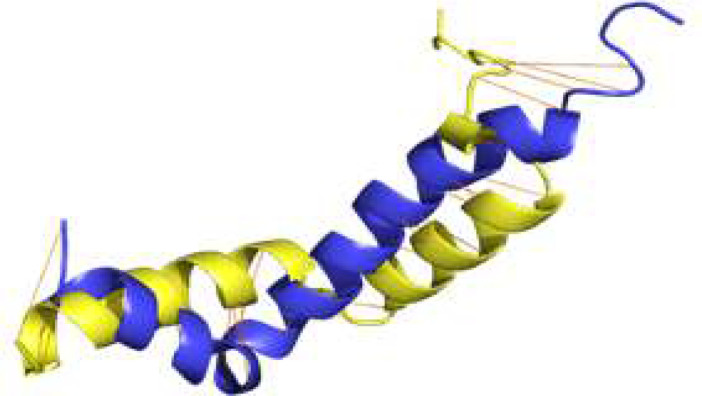
The structures of Aβ from *H. sapiens* and *H. glaber* were aligned by PyMOL; orange lines indicate the efficiency of the alignment. The yellow and blue structures belong to *H. glaber* and *H. sapiens*, respectively

Furthermore, we tried recycling numbers in the range of 3 to 24 [Fig. 3 a-d] and we encountered the highest pTM and pLDDT values from recycling number 12, which determines it as the proper recycling number [Fig. 3c and Table S3]. For further investigation, we aligned our model with *H. sapiens* Aβ peptide (PDB ID: 1IYT) in the PyMOL. Orange lines are the alignment confidence [Fig. 4]. The RMSD value between two molecules is 7 angstroms. The structure is similar to the nuclear magnetic resonance structure of the *H. sapiens* Aβ. The difference in the Arg13 did not affect the 3D structure of Aβ backbone conformation in any significant way [Fig. 4], indicating that the *H. glaber* Arginine took part in the formation of alpha helix just as the *H. sapiens* Histidine did.

β

**Figure 5:**
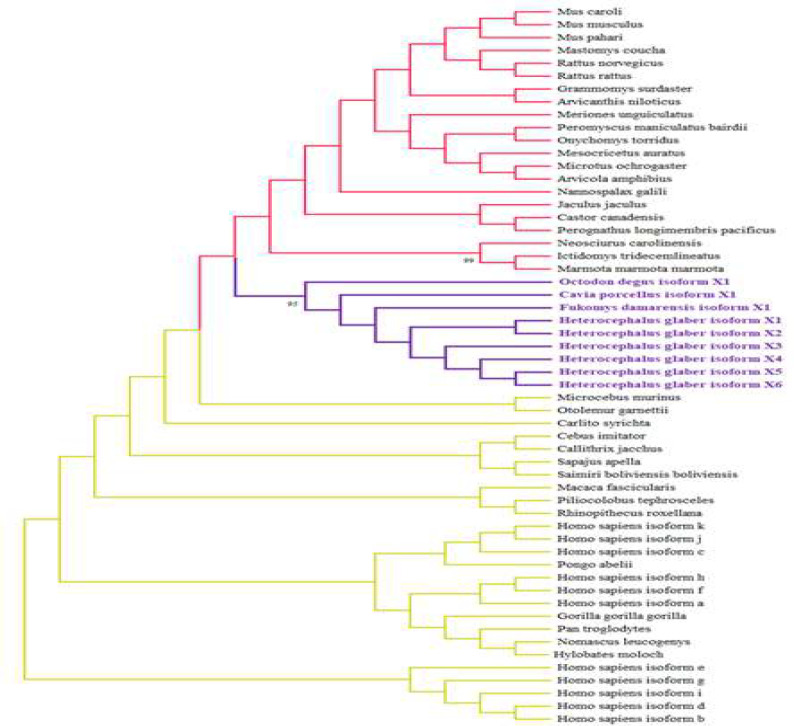
The evolutionary tree is inferred from the APP sequences of taxa Primata and Rodentia. The Maximum Likelihood technique and JTT matrix-based model were used to estimate the evolutionary history. The evolutionary history of the species under study is represented by the bootstrap consensus tree generated from 1000 repetitions. Branches associated with partitions that were replicated in fewer than 50% of bootstrap replicates are collapsed. 57 amino acid sequences were examined in this investigation. The MEGA11 software conducted evolutionary analyses. The clades of rodentia and primata are colored red and yellow, respectively. The purple color shows the valid clade

The phylogenetic tree was obtained with APP sequences as inputs using the Mega11 software. All the APP isoform X1s of primates and rodents were utilized for generating the phylogenetic tree. The tree was generated by the maximum likelihood algorithm. According to the previous investigations of several proteins in rodents, the same clades of likelihood were acquired, as demonstrated in our phylogenetic tree of the Aβ precursor protein. Species *H. glaber*, *Fukomys damarensis*, and *Cavia porcellus* are closest, and *Octodon degus* is also in the same clade [37, 38] indicating that all these rodents are closely related. Notably, due to the high bootstrap number, 95 for this clade, the data is reliable [Fig. 5]. The tree shows that primates are on a completely separate clade. These observations show that APP sequences are originally different among the two taxa yet very conserved among species in each taxon. Regarding rodents, *H.glaber*, *F.damarensis*, *C.porcellus*, and *O.degus* are shown in the same clade indicating a close homology between their precursor proteins. Sequences of APP in rodentia were highly conserved, and every genus was grouped together; however, the clade accuracy number was not high enough. *H. sapiens* APP sequences were almost highly conserved, but the phylogenetic tree showed that they are not all placed in only one clade, which shows that the similarities were high among other primates and *H. sapiens*.

Furthermore, comparing the Aβ sequences region in different isoforms of *H. glaber* shows that the 42 amino acids are fully conserved. The same goes for the *H. sapiens* Aβ peptides region derived from different isoforms, with all of them being 100% conserved [Fig. S2]. We want to point out that APP is the integral protein, which turns into Aβ peptides by the cleavage of its extracellular domain. Our main focus has been on Aβ peptide throughout this project since it is the main component of fibrils and plaques found in the brain in Alzheimer’s disease. Yet to capture the most diversity, the full APP sequences were used in the Phylogenetic analysis.

**Figure 6:**
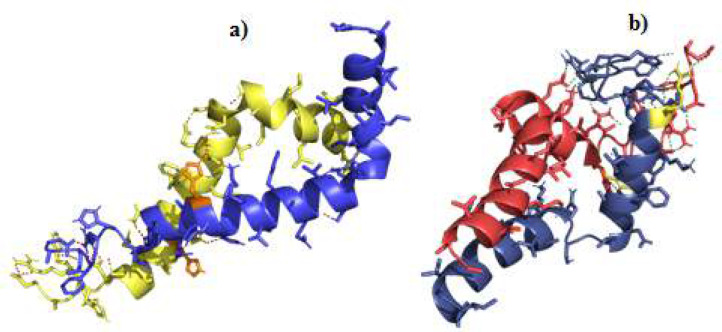
Structure presentation molecular docking results of *H. sapiens* Aβ peptides and *H. glaber* Aβ peptides in **(a)** and **(b)** respectively. Aβ peptides of *H. sapiens* are blue and yellow and His13s are illustrated in orange color. Aβ peptides of *H. glaber* are dark blue and red and Arg13s are shown in yellow color. All intramolecular and intermolecular interactions within 4.0 angstroms are shown with red **(a)** and green **(b)** dashes

**Figure 7:**
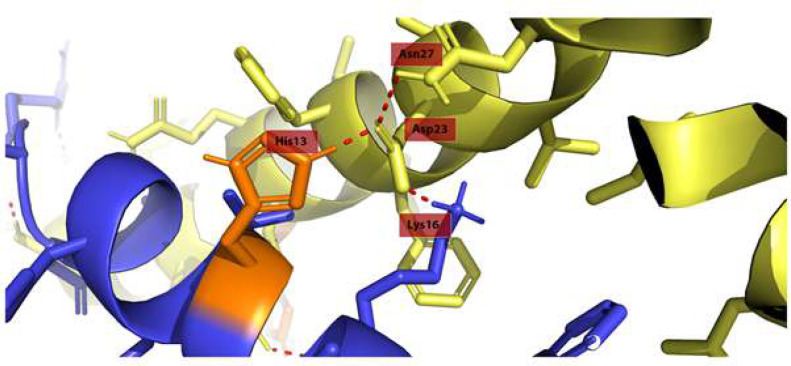
Inter- and intramolecular interactions of *H. sapiens* AB peptide, highlighting the role of His13 in formation of bonds in dimer structure. Red lines depict molecular interactions and the name of each residue is mentioned in a red box near it

Docking was conducted by HADDOCK 2.4 between two peptide structures of both *H. sapiens* (PDB ID: 1IYT) and the AlphaFold2-predicted Aβ peptide of *H. glaber* to investigate the aggregation mechanism of the Aβ in both organisms. The first HADDOCK clusters were selected for both species because of their best scores. Radius of gyration for these clusters were calculated. *H. glaber’s* radius of gyration was 15 angstroms, while this score for *H. sapiens *was 17 angstroms. The score is lower for *H. glaber* which indicates that the structure is more compact and atoms are closer to the peptide’s center of mass; thus, it may adapt a globular structure in comparison to *H. sapiens*’ Aβ structure. *H. sapiens* Aβ docking results showed potential for fibril formation [Fig. 6a], thus ultimately resulting in plaque formation as a hallmark of AD, but *H. glaber* Aβ [Fig. 6b] cannot form the fibrillar structure because of a globular conformation seen in the docking results. In the *H. sapiens* structure, Asp23 interacts with Asn27 in the same peptide and His13 and Lys16 of the other [Fig. 7]. However, according to the docking results, none of the mentioned amino acids in the structure of *H. glaber* Aβ create interactions with the amino acids from the other peptide [Fig. 6b].

HADDOCK scores were -82 for *H. sapiens* and -78 for *H. glaber*. For *H. glaber* dimer formation, the electrostatic energy was -157 kcal.mol^-1^; meanwhile, *H. sapiens* conformation electrostatic energy was twice as much as *H. glaber*, and it was -310 kcal.mol^-1 ^[Table 1]. This difference accounts for structural stability, which is higher for *H. sapiens* Aβ and depicts more potential for plaque formation. This shows that the conformational change caused by one amino acid difference H13R in these two species, leads to the structure instability in *H. glaber* Aβ.

**Table 1:** Molecular docking data of two Aβ peptides together represented by HADDOCK outputs

**No.**	**Haddock** **score**	**Cluster** **size**	**RMSD**	**Vdw** **energy**	**Elect** **energy**	**Desol** **energy**	**Restr** **energy**	**Buried surface area**	**Z-score**
HS									
1	-82.4 ± 7.8	9	21.3 ± 0.2	-43.1 ± 5.8	-310.6 ± 60.0	-32.9 ± 6.1	557.4 ± 75.6	2008.5 ± 103.0	-1.2
2	-81.4 ± 26.6	4	21.3 ± 0.4	-41.3 ± 5.0	-346.6 ± 105.1	-31.2 ± 6.4	604.5 ± 137.2	1992.2 ± 191.1	-1.0
3	-75.5 ± 18.0	4	20.3 ± 0.3	-55.5 ± 7.5	-157.3 ± 40.0	-42.0 ± 2.9	535.0 ± 124.5	2120.9 ± 53.2	-0.2
4	-74.9 ± 14.5	6	20.6 ± 0.2	-41.3 ± 8.0	-303.4 ± 58.5	-28.4 ± 3.4	554.5 ± 62.7	1995.7 ± 63.8	-0.1
5	-69.6 ± 13.3	4	21.3 ± 0.3	-44.3 ± 11.8	-231.7 ± 45.8	-29.3 ± 9.8	502.7 ± 39.6	2078.6 ± 102.3	0.7
6	-62.3 ± 9.3	8	10.2 ± 0.3	-52.0 ± 5.7	-139.2 ± 21.2	-36.8 ± 5.1	543.9 ± 100.4	2035.6 ± 119.1	1.8
HG									
1	-78.1 ± 5.0	7	6.0 ± 0.6	-63.7 ± 3.9	-157.7 ± 33.0	-31.0 ± 4.8	481.1 ± 79.4	2293.7 ± 156.9	-1.5
2	-77.8 ± 13.4	21	8.2 ± 0.4	-58.8 ± 10.5	-244.6 ± 53.5	-14.6 ± 4.4	445.1 ± 37.5	2221.9 ± 104.3	-1.4
3	-76.6 ± 5.6	5	5.3 ± 0.9	-60.5 ± 1.7	-242.4 ± 13.7	-16.6 ± 7.9	490.1 ± 120.8	2446.7 ± 52.0	-1.2
4	-69.7 ± 15.0	7	8.0 ± 0.6	-49.1 ± 10.6	-203.6 ± 12.1	-27.9 ± 3.3	480.4 ± 98.8	2074.4 ± 157.8	0.0
5	-68.7 ± 17.1	5	17.1 ±0.2	-63.3 ± 5.5	-93.7 ± 30.1	-30.3 ± 3.1	436.1 ± 46.9	1964.8 ± 104.4	0.1
6	-67.3 ± 12.4	4	7.2 ± 0.5	-30.2 ± 8.8	-202.3 ± 62.6	-40.9 ± 5.3	442.7 ± 86.0	2369.1 ± 79.5	0.4
7	-67.1 ± 11.8	7	6.7 ± 0.6	-46.3 ± 11.1	-201.1 ± 31.4	-29.6 ± 9.0	489.3 ± 44.9	2193.7 ± 139.7	0.4
8	-66.6 ± 16.3	4	4.9 ± 0.3	-52.6 ± 11.8	-160.9 ± 29.1	-28.3 ± 3.1	465.2 ± 60.9	2221.1 ± 153.4	0.5
9	-63.9 ± 5.7	9	8.0 ± 0.6	-54.0 ± 5.8	-240.4 ± 47.0	-17.5 ± 3.9	556.8 ± 35.4	2150.0 ± 77.0	1.0
10	-60.0 ± 17.4	8	11.2 ± 0.2	-49.0 ± 6.6	-149.0 ± 28.2	-28.8 ± 3.4	475.2 ± 99.0	1890.6 ± 98.1	1.7

**Footnote:** The table starts with *H. sapiens* (HS) molecular docking results and is continued by *H. glaber* (HG) results.

**Figure 8:**
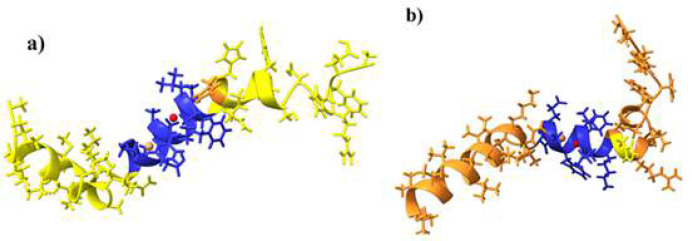
Demonstration of Aβ peptide, Zn^2+^, and Cu^2+^ ions molecular docking results of *H. sapiens* Aβ peptide and *H. glaber* Ab peptide in **(a)** and **(b)**. *H. sapiens* Aβ **(a)** is yellow colored, and His13 is shown in orange in the structure. *H. glaber* Aβ **(b)** is orange and Arg13 is shown in yellow in the structure. The binding sites of ions are illustrated with blue color in the helix

Further docking of *H. glaber* Aβ peptide with Zn^2+^ and Cu^2+^ ions has predicted the binding sites of these ions to be situated within 4.0 angstroms of residues Lys16 to Asp23, which is not in direct contact with Arg13. The same ions were located further apart from each other when docked with *H. sapiens* Aβ peptide and within 4.0 angstroms of residues Lys16 to Ser26, which also happens to be not in direct contact with His13. Despite no apparent connection between these residues, the Asp23 in *H. sapiens* is in the range of the Zn^2+^ binding site of one strand and His13 of the other Aβ peptide strand [Fig. 8].

According to the sequence alignment results we noticed that the Aβ sequence of *Marmota marmota* is different from the one of *H. sapiens* in amino acids 10 and 13, and from the one of *H. glaber* only in the 10th residue (Fig. S2). Therefore, to further address the novelty of our work, we extracted the Aβ sequence of *M. marmota* from the APP sequence [Acc No: XP015334152.1]. We predicted the 3D structure of the Aβ peptide with AlphaFold2 and then used it to perform self-docking with HADDOCK 2.4. The test results are available in supplementary information [Table S.4 and S.5]. After visualization of the docking results by PyMOL, we found out that the two docked *M. marmota* Aβ peptides formed a more stable and more fibrillar dimer compared to *H. glaber* (Fig. S3b). This strongly suggests that *M. marmota* is more likely to form fibrils and plaques and could be more prone to Alzheimer’s disease compared to *H. glaber*. Moreover, by comparing the electrostatic energy values of Aβ dimers formed in *H. glaber*, *M. Marmota*, and *H. sapiens* (-150, -200, -300 kcal/mol respectively), it can further be concluded that *M. marmota* forms dimers that are more stable than the ones in *H. glaber*, yet less stable compared to dimers formed in *H. sapiens*.

## DISCUSSION

Aβ has a crucial role in AD, specifically, Aβ 1-42, considering its strong aggregation propensity [39]. Given the difficulty in identifying clinically relevant biomarkers of AD progression, novel criteria, such as specific molecular polymorphic forms of Aβ, need to be examined [40]. APP, the integral protein from which Aβ peptide is derived, has remained remarkably conserved throughout evolution; primates, dogs, and bears have shown signs of cerebral amyloid deposits in the form of fibrils. However, there is no notable record of such deposits in the brains of mice and rats [13]. Understanding the differences between the structural properties of Aβ in primates and rodents can aid us in finding the reasons behind the rodents' immunity to AD. Our findings indicate that the Arg13’s side chain in the *H. glaber*’s Aβ sequence and the His13’s side chain in the primates Aβ sequence are placed with different angles in comparison to one another and this deviation can play an important role when it comes to the peptide’s tendency to self-aggregate.

Our phylogenetic analysis exhibits remarkable homology between APP sequences in rodents. *H. glaber* was placed in the same clade as *F. damarensis* (*Damara mole-rat*), *C. porcellus* (*Guinea pig*), and *O. degus* (*Common degu*). Further studies showed that *H. glaber*, *F. damarensis*, and *O. degus* all express the same Aβ sequence, which differs from the *C. porcellus’* sequence in only one residue (R13H) [41-43]. Studies have shown that the His13 is of great importance regarding the aggregation properties and neurotoxicity of the Aβ peptide. Substitution of the previously mentioned His with Arg diminishes the peptide’s affinity for metal ions such as Zn^2+^, which reduces Aβ’s aggregation propensity and toxicity, since the side chain of His13 is a coordination site for Zn^2+^. Studies have also indicated that Aβ peptide does not aggregate significantly in the absence of metal ions. Additionally, His13 residue in *H. sapiens* Aβ is involved in β-sheet formation and methylation of the peptide [17, 44]. This supports our findings about *H. sapiens* having a stronger affinity for aggregation of Aβ dimer complexes.

Although *H. glaber* tolerates high Aβ levels without showing any signs of plaque formation [45], expressing the same Aβ as *H. glaber* [23, 45-49], *O. degus* has shown signs of diffuse Aβ accumulations as well as cognitive decline. Aged degus, in particular, have been observed to develop molecular symptoms of AD, such as an increase in Aβ peptides and production of phosphorylated tau [45, 50]. *C. porcellus*, with an Aβ sequence identical to *H. sapiens*, also shows age-related signs of hippocampal Aβ deposits and Aβ oligomers [41, 42, 50]. However, the presence of senile plaques has not been reported in any of the rodents mentioned above [6].

Since Aβ peptides in primates self-aggregate [39], we reasoned that docking these peptides obtained from AF2 could lead to a better understanding of plaque-forming affinity. Molecular docking of two Aβ peptide structures together exhibited that *H. sapiens* Aβ peptides have a great tendency to self-aggregate. Whereas, the *H. glaber* Aβ peptides are less likely to form a dimer. The replacement of the His13 with Arg draws the backbones of the *H. glaber* peptides away from each other in comparison to the peptides in *H. sapiens*. This change of placement can effectively reduce the probability of forming oligomers, protofibrils, fibrils, and, subsequently, amyloid plaques. Our findings can explain the plausible reason behind *H. glaber*’s immunity to plaque formation and Alzheimer’s disease.

Comparing the results from *H. glaber*, *H. sapiens* and *M. marmota* brings up the conclusion that it is not crucial how many amino acids are different but rather the type of the amino acid itself, its position, its interactions, and its surrounding amino acids are better determinants of the final structure and behavior of the peptide or protein in the environment it’s found in. We acknowledge that one amino acid difference is not a huge difference that could make a noticeable alteration in Aβ molecular assembly, but it can alter the whole plaque formation mechanism.

Furthermore, the molecular docking of *H. sapiens* and *H. glaber* Aβ structures with Zn^2+^ and Cu^2+^ ions determined that even though the His13 in *H. sapiens* and Arg13 in *H. glaber* are not in the binding sites of these ions, they are in contact with the other strand’s metal binding site when a dimer of two Aβs is formed. This finding can not only confirm the speculated Zn^2+^ and Cu^2+^ binding site placement in other researches to be accurate [17], but also shows that the substitution of His13 with Arg13 can play a role in the plaque formation affinity between two Aβ strands.

However, focusing on Aβ alone as an element initiating Alzheimer’s disease may not be legitimate. Even though Aβ is the superstar of dementia, other proteins are also involved in neurodegenerative diseases, such as cystatin, transthyretin, and the British and Danish types of amyloid these proteins accumulated in the brain and vessels in a way that they can also affect brain function [51].

Although it has been proposed previously that the self-aggregation of Aβ peptides includes a stage of conformational change from alpha helices to β-strands, we didn't encounter any evidence in our findings showing that. Beta structures are overall less likely to happen; however, regions 10-30 and 35-38 have, on average, greater than a 20% chance of β-strand formation [52]. Our Rank4 prediction includes β-strands in residues 17-23 and 30-36. These strands are about 70 to 90 percent confident, according to AlphaFold pLDDT, and the PAE plot shows low relative position accuracy. We hope that our recent findings help others with a further understanding of the relationship between Aβ structure and Alzheimer's disease.

### Conflict of Interest:

The authors declare no competing interests.

### Authors’ Contribution:

MAI designed the study and supervised the project, contributed in interpretation of the results and edited the manuscript. AJ carried out AlphaFold2 analyses, conducted phylogenetic analyses, carried out molecular dockings, wrote the methods, results and conclusion, organized the final frame of the manuscript, provided the tables 1, S.4 and S.5, figures 1-4, 6, 7, S.2 and S.3 and designed the graphical abstract. GT carried out AlphaFold2 analyses, carried out molecular dockings, contributed in writing abstract, introduction and discussion. AE carried out AlphaFold2 analyses, conducted phylogenetic analyses, contributed in writing abstract, methods, results and conclusion, provided figure 5, tables S.1, S.2 and S.3 and designed the graphical abstract. MSK carried out AlphaFold2 analyses, and wrote introduction and discussion. SMHH provided figures 8 and S.1 and contributed to facilitating the computational technicalities in AlphaFold2, PyMOL and ChimeraX. All authors read the manuscript thoroughly.

## Supplementary Materials


