# Supplementary material for: In-silico structural analysis of Heterocephalus glaber amyloid beta: an anti-Alzheimer's peptide
Source: Mol Biol Res Commun. 2024;13(1):29–42. doi: 10.22099/mbrc.2023.48223.1862 (PMC10644309; doi:10.22099/mbrc.2023.48223.1862)
Supplement: Supplementary file 1 [file mbrc-13-29.s001.pdf]

Table S1: Accession numbers of APP sequences of rodentia and primata.

Accession numbers	Species
XP 021040427.1	Mus caroli
NP 001185752.1	Mus musculus
XP 021064992.1	Mus pahari
XP 031220168.1	Mastomys coucha
NP 062161.1	Rattus norvegicus
XP 032756378.1	Rattus rattus
XP 028613034.1	Grammomvs surdaster
XP 034371116.1	Arvicanthis niloticus
XP 021514015.1	Meriones unguiculatus
XP 042115444.1	Peromvscus maniculatus bairdii
XP 036060101.1	Onychomvs torridus
XP 040606481.1	Mesocricetus auratus
XP 005345348.1	Microtus ochrogaster
XP 038201642.1	Arvicola amphibius
XP 017657176.1	Nannospalax galili
XP 004654437.1	Jaculus jaculus
XP 020041696.1	Castor canadensis
XP 048202323.1	Perognathus longimembris pacificus
XP 047420615.1	Neosciurus carolinensis
XP 040143139.1	Ictidomvs tridecemlineatus
XP015334152.1	Marmota marmota marmota
XP 004627753.1	Octodon degus
XP 003467233.1	Cavia porcellus
XP 010606866.1	Fukomys damarensis
XP 004842285.1	Isoform X1 Heterocephalus glaber
XP 021111788.1	Isoform X2 Heterocephalus glaber
XP 004842288.1	Isoform X3 Heterocephalus glaber
XP 021111789.1	Isoform X4 Heterocephalus glaber

XP 004842293.1	Isoform X5 Heterocephalus glaber
XP 021111790.1	Isoform X6 Heterocephalus glaber
XP 012619905.1	Microcebus murinus
XP 023365467.1	Otolemur garnettii
XP 008048131.1	Carlito svrichta
XP 017397394.1	Cebus imitator
XP 002761374.2	Callithrix jacchus
XP 032132996.1	Sapajus apella
XP 039336411.1	Saimiri boliviensis boliviensis
XP 005548940.1	Macaca fascicularis
XP 023045769.1	Piliocolobus tephrosceles
XP 010381738.1	Rhinopithecus roxellana
NP 001372182.1	Isoform k Homo sapiens
NP 001191232.1	Isoform j Homo sapiens
NP 958817.1	Isoform c precursor Homo sapiens
NP 001127014 1	Pongo abelii
NP 001191230.1	Isoform h Homo sapiens
NP 001129602.1	Isoform f Homo sapiens
NP 000475.1	Isoform a Homo sapiens
XP 004062681.1	Gorilla gorilla gorilla
NP 001013036.1	Pan troglodytes
XP 030662027.1	Nomascus leucogenys
XP 032030716.1	Hvlobates moloch
NP 001129601.1	Isoform e precursor Homo sapiens
NP 001129603.1	Isoform g Homo sapiens
NP 001191231.1	Isoform i Homo sapiens
NP 001129488.1	Isoform d Homo sapiens
NP 958816.1	Isoform b Homo sapiens

Table S2: The pTM and mean pLDDT values of different *H. glaber* predicted models.

Predicted Model	Mean pLDDT Value	pTM Value
Rank 1	81.5	0.46
Rank 2	76.0	0.31
Rank 3	75.8	0.35
Rank 4	67.6	0.3
Rank 5	60.3	0.17

Table S3: Mean pLDDT and pTM values of different recycling numbers.

Predicted Model	Mean pLDDT Value	pTM Value
Recycle number 3	70.9	0.26
Recycle number 6	81.2	0.46
Recycle number 12	81.5	0.46
Recycle number 24	80.8	0.46

Table S4: Molecular docking data of *M. Marmota* Aβ peptides dimer represented by HADDOCK outputs.

No.	Haddock score	Clus ter size	RMSD	Vdw energy	Elect energy	Desol energy	Restr energy	Buried surface area	Z-scor e
1	-106.4 ± 10.4	21	2.3 ± 1.4	-68.9 ± 8.6	-194.1 ± 41.1	-30.6 ± 3.5	319.6 ± 12.7	2211.8 ± 54.9	-2.0

Table S5: The pTM and mean pLDDT values of different *M. marmota* predicted models.

Predicted Model	Mean pLDDT Value	pTM Value
**Rank 1**	**68**	**0.28**
**Rank 2**	**53**	**0.14**
**Rank 3**	**52**	**0.16**
**Rank 4**	**49**	**0.14**
**Rank 5**	**48**	**0.14**

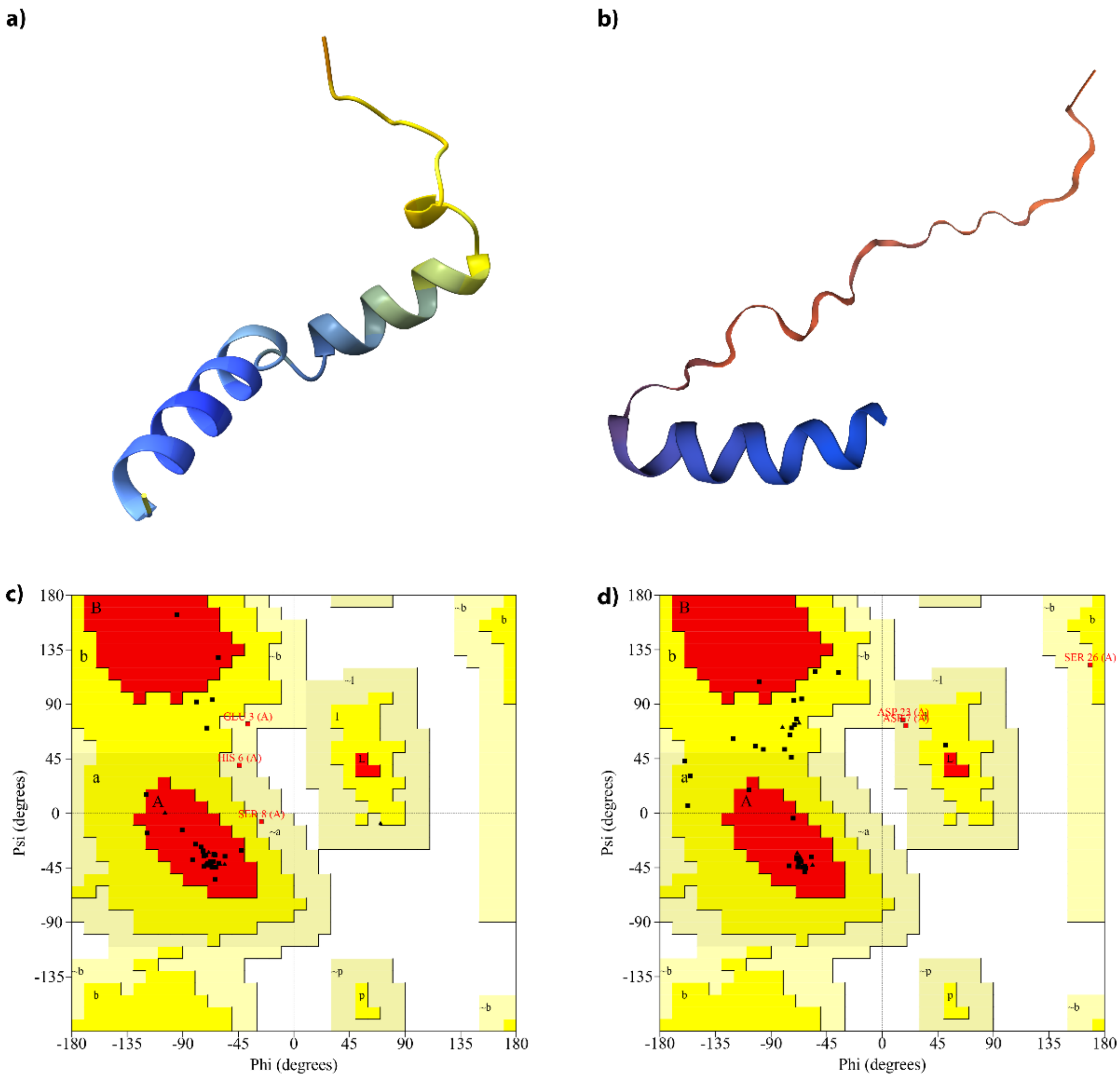

Figure S1: The Ramachandran plot of AlphaFold2 and SWISS-MODEL predicted Aβ peptides generated by PROCHECK.

The 3D structure of *H. glaber* Aβ peptide predicted by AlphaFold2 and SWISS-MODEL are visualized in a and b, respectively. There are four regions in the Ramachandran plots that include: Most favored (red), Additional allowed (yellow), Generously allowed (light yellow), and disallowed (white). c) The AlphaFold2 Aβ peptide plot shows that 82.4% of residues are in the most favored region. The additional allowed and generously allowed contain 8.8% and 8.8% of the residues, respectively. d) The SWISS-MODEL Aβ peptide plot shows that 41.2% of residues are in the most favored region. The additional allowed and generously allowed contain 50.0% and 8.8% of the residues, respectively.

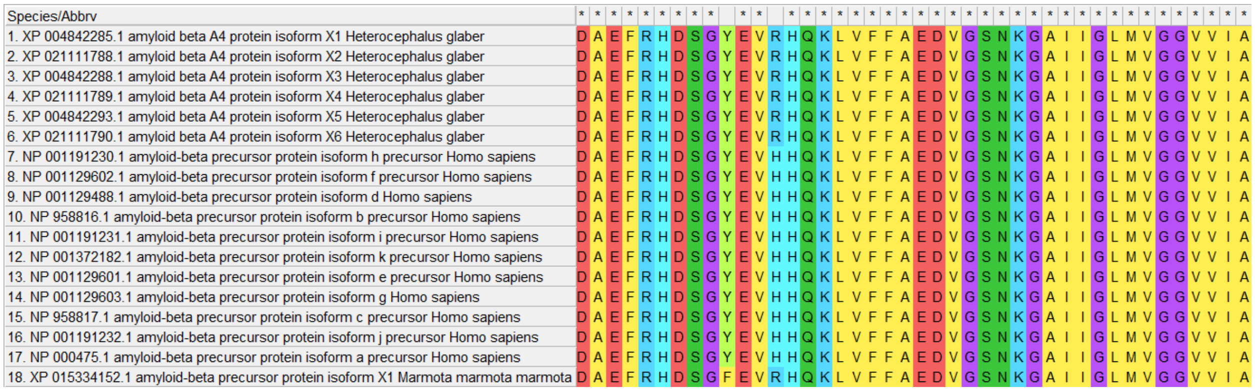

Figure S2: Aβ sequence alignment of *H. glaber*, *H. sapiens,* and *M. marmota*.

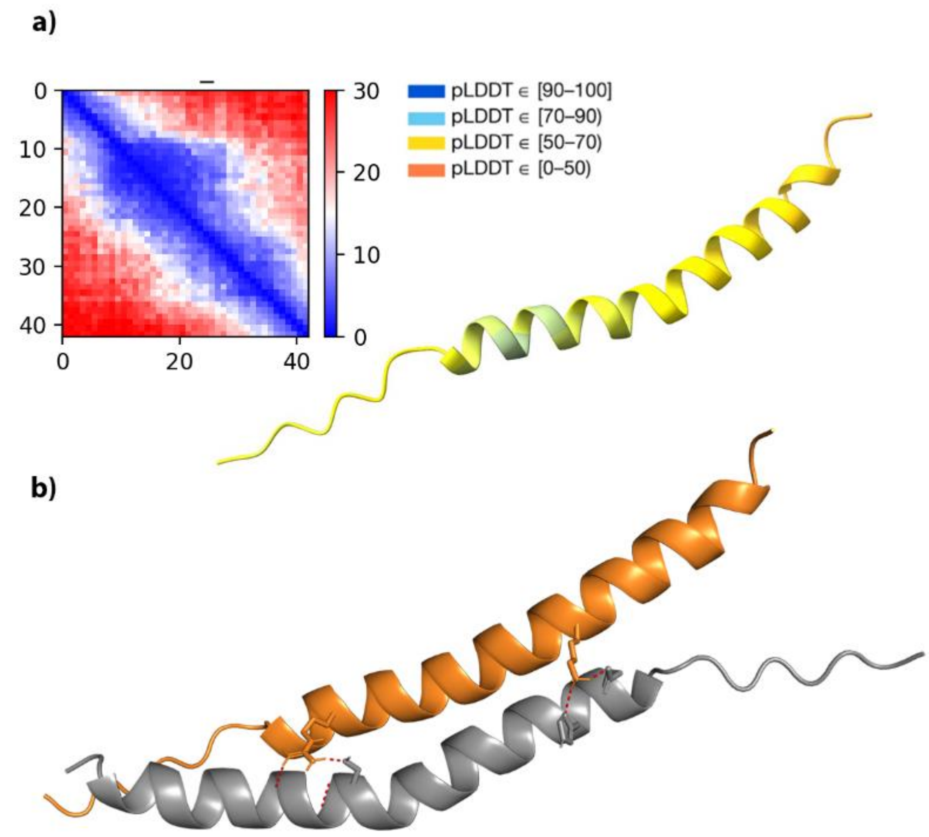

Figure S3: *M. marmota* Aβ sequence structure prediction and inspection of dimer formation. a) Structure of *M. marmota* Aβ predicted by AlphaFold2. The Aβ structure is colored by the pLDDT coloring score and the PAE plot is showing the accuracy of relative positions. b) Aβ dimer conducted by HADDOCK. Red lines show intermolecular interactions between these two Aβ dimers.
